# Subjective executive functioning and skill learning during the COVID-19 pandemic associated with perceived loneliness, depressive symptoms, and well-being

**DOI:** 10.1371/journal.pmen.0000372

**Published:** 2025-07-31

**Authors:** Lilian Azer, Isaac Quintanilla Salinas, Esra Kürüm, Leah Ferguson, Elizabeth L. Davis, Weiwei Zhang, Carla M. Strickland-Hughes, Rachel Wu

**Affiliations:** 1 Department of Psychology, University of California, Riverside, California, United States of America; 2 Department of Neurobiology and Behavior, University of California, Irvine, United States of America; 3 Department of Statistics, University of California, Riverside, California, United States of America; 4 Department of Psychology, University of the Pacific, Stockton, California, United States of America; University of Padova: Universita degli Studi di Padova, ITALY

## Abstract

The present studies investigated whether higher subjective executive functioning and learning new skills related to better mental health across adulthood during the COVID-19 pandemic. Participants (n = 133) were recruited from the Inland Empire in Southern California, USA across two different timepoints. A subset of participants over the age of 58 years old, who previously participated in a skill learning intervention (*n* = 52) that increased objective executive functioning, also were included in the present studies. Worse subjective executive functioning (EF) during the COVID-19 pandemic predicted worse mental health across adulthood. In addition, learning new skills may have helped adults adapt better to changes during the pandemic to increase mental health. These findings highlight the importance of cognitive abilities and learning new skills on mental health. Our findings provide a more nuanced view of the benefits and costs of adaptation via skill learning on adult mental health during the COVID-19 pandemic. Learning new skills could be associated with better mental health outcomes and we see this during the second timepoint of the COVID-19 pandemic. In addition, worse subjective cognitive abilities could lead to worse mental health outcomes. Our results suggest that better cognitive abilities and learning new skills are important for mental health.

COVID-19 physical distancing restrictions exacerbated perceived loneliness, depressive symptoms, anxiety, and lowered overall levels of well-being in adults [1–8]. Some studies have reported that younger adults felt lonelier and experienced more negative emotions and worse psychological well-being during the COVID-19 pandemic, compared with older adults [9,10] However, when longitudinal data were collected, Luchetti and colleagues (2020) found that younger adults felt more lonely during the initial stage of the COVID-19 pandemic, but over time, older adults’ perceived loneliness increased compared with younger adults’ due to an increased sense of diminished social connections. Mental health outcomes throughout the duration of the pandemic have been extensively studied. However, executive functioning and skill learning in relation to mental health outcomes has yet to be investigated across different time points during the COVID-19 pandemic. Therefore, the present studies investigated the relationship between executive functioning and skill learning on mental health during the pandemic across several time points.

Studies examining mental health outcomes during the COVID-19 pandemic have rarely how subjective cognitive abilities (i.e., self-reported cognitive function) and novel skill learning may have helped, or hindered, adults’ ability to cope with changes during the pandemic. Research prior to the COVID-19 pandemic suggested that better subjective cognitive ability may be an important factor that could influence lower levels of loneliness, depressive symptoms, and higher levels of overall well-being, especially among older adults [11–13]. In particular, across these studies pre-pandemic worse subjective executive functioning (self-reported working memory, problem solving, monitoring, etc.) was associated with increased loneliness and depressive symptoms and worse overall well-being. Executive functioning involves multiple cognitive processes, including cognitive control and working memory, and often declines in normative aging [14–16]. Self-reported decline in executive functioning (increased cognitive complaints) is related to objective cognitive decline (measurable cognitive function using a memory test; [17,18]. In addition, self-reported decline in executive functioning may also predict Alzheimer’s disease symptomatology later in life [19]. By including a more diverse population compared to pre-pandemic studies, the present studies aimed to provide a better understanding of the impact of the COVID-19 pandemic on cognitive abilities, learning, and mental health.

In addition to the relationship between mental health outcomes and cognitive functioning, engaging in leisure activities, specifically learning new skills, is also associated with cognitive functioning (see the enrichment hypothesis, [20]. Studies pre-pandemic showed that learning new skills increased executive functioning in adults (e.g., [21], and was related to better mental health during stressful times [22–25]. Indeed, during the pandemic, engaging in leisure activities, which may include novel skill learning, seemed to have that benefit [26]. Novel skill learning may make stressful situations like the pandemic more tolerable, especially if the skill fills a functional need (e.g., learning to use tech devices and software for remote options).

Studies pre-COVID-19 pandemic found that better executive functioning can positively impact mental health outcomes [11–13], and learning new skills also can improve mental health outcomes [22–25]. However, the relationship between executive functioning and learning new skills on mental health outcomes during the COVID-19 pandemic has not been as robust as the results reported prior to the COVID-19 pandemic across older and younger adults [9,10,27,28]. Time point of data collection during the COVID-19 pandemic could have contributed to these mixed findings. For instance, when mental health was assessed during a single time point during the first few months of the COVID-19 pandemic, younger adults reported feeling lonelier and having worse well-being compared to older adults [9,10]. However, when loneliness was assessed longitudinally during the COVID-19 pandemic, younger adults reported feeling increased loneliness during the initial stage of the pandemic compared to older adults but as time progressed older adults’ perceived loneliness increased compared with younger adults [27].

In addition to the inconsistencies across these mixed cross-sectional findings, longitudinal studies examining changes in mental health outcomes from pre-COVID-19 pandemic found initial declines in mental health outcomes from pre-pandemic to the first time point of data collection [29–32]. However, these studies did not find a significant difference in mental health outcomes across the different time points of data collection during the COVID-19 pandemic. Similarly, longitudinal studies assessing mental health outcomes throughout the duration of the pandemic found that the peak of mental health decline occurred in the early months of the pandemic and there were no significant changes in mental health outcomes when tested across multiple time points during the pandemic [33,34]. One possibility could be that as the pandemic progressed, individuals adapted to the new restrictions, which had a less severe impact on their mental health over time. In fact, individuals who lived in areas where COVID-19 restrictions were more stringent experienced greater psychological distress in the early months of the COVID-19 pandemic [31]. Thereafter, changes in mental health across the 15 months data collection period were minimal.

## The present studies

The present studies investigated whether subjective executive functioning and novel skill learning in adults predicted mental health outcomes (perceived loneliness, depressive symptoms, and overall well-being) across two time points during the COVID-19 pandemic. In addition to community members recruited for the present studies, we investigated this relationship using a unique population: a group of adults who participated in a prior cognitive learning intervention that increased objective executive functioning [21] in order to assess involvement in a skill learning intervention on mental health outcomes during the COVID-19 pandemic. Older adults who participated in the cognitive learning intervention learned at least three new skills in a classroom setting (i.e., Spanish classes, learning to use an iPad, etc.) over a 2-to-3-month period and participated in weekly discussions about successful aging, growth mindset, and motivation for learning. The intervention included a pre-assessment of cognitive abilities (working memory, cognitive control), followed by in-person, 2-hour classes focused on real-world skill learning (e.g., photography, Spanish, using an iPad, music composition, painting/drawing) each week. Some participants learned only one skill for two hours a week, while others learned 3 or even 5 skills per week. For the participants who completed the intervention, they took these classes for 3 months. However, many of the intervention participants were part of the group that had an intervention cancelled due to COVID restrictions in Week 8, two-thirds of the way through the program. Participants were assessed periodically before and after the end of the intervention. In two groups, Leanos and colleagues found that the intervention participants’ objective executive functioning increased to baseline levels of middle-aged adults (30 years younger) by the end of the intervention, and to younger adult levels (50 years younger) at the 1-year follow-up [35]. For more details on the intervention tasks and outcomes, see Leanos et al. (2023).

Across two studies, there were three primary mental health outcomes, perceived loneliness, depression, and overall well-being. For both Studies, we hypothesized that 1) better self-reported executive functioning would predict better mental health during the pandemic across all adults, 2) increased age would predict worse mental health outcomes, and 3) learning new skills during the pandemic would predict better mental health outcomes. In addition, given the inconsistencies in the literature, we also explored how mental health for our participants changed during the different time points of data collection.

While both studies were interested in examining similar predictors on mental health outcomes with similar hypotheses, two distinct studies were included in our analyses to isolate the potential effects of the cognitive learning intervention on these outcomes. That is, Lenos et al. (2023) and Ferguson et al. (2023) demonstrated that participation in the intervention significantly enhanced cognitive functioning in older adults, with these improvements continuing at a one-year follow-up. Therefore, to avoid confounding the general population analyses with these known intervention effects, we excluded intervention participants from Study 1. Study 1 therefore focused on a general, non-intervention sample. In contrast, Study 2 specifically included participants from the cognitive learning intervention in a specific age range, allowing us to examine whether the intervention influenced mental health outcomes directly or interacted with self-reported cognitive functioning to predict mental health outcomes for middle-aged and older adults. Therefore, in Study 2 we predicted that those who participated in the cognitive intervention would have decreased feelings of perceived loneliness and depression, and overall better well-being compared with those who did not participate in the intervention.

The studies reported in this article were not formally preregistered. Neither the data nor the materials have been made available on a permanent third-party archive; data is available at https://doi.org/10.5061/dryad.69p8cz9ff; requests for materials can be sent via email to the corresponding author.

## Method

### Transparency and openness

Sample size determination and all data exclusions were reported in the study. The study design, hypotheses, and analytic plan were not preregistered. All data are available on Dryad: ( [36]; https://doi.org/10.5061/dryad.69p8cz9ff).

### Ethics statement

All research procedures were approved by the Institutional Review Board (20–082) at the University of California, Riverside, and all participants were provided written informed consent.

### Participants (studies 1 and 2)

**Fig 1:**
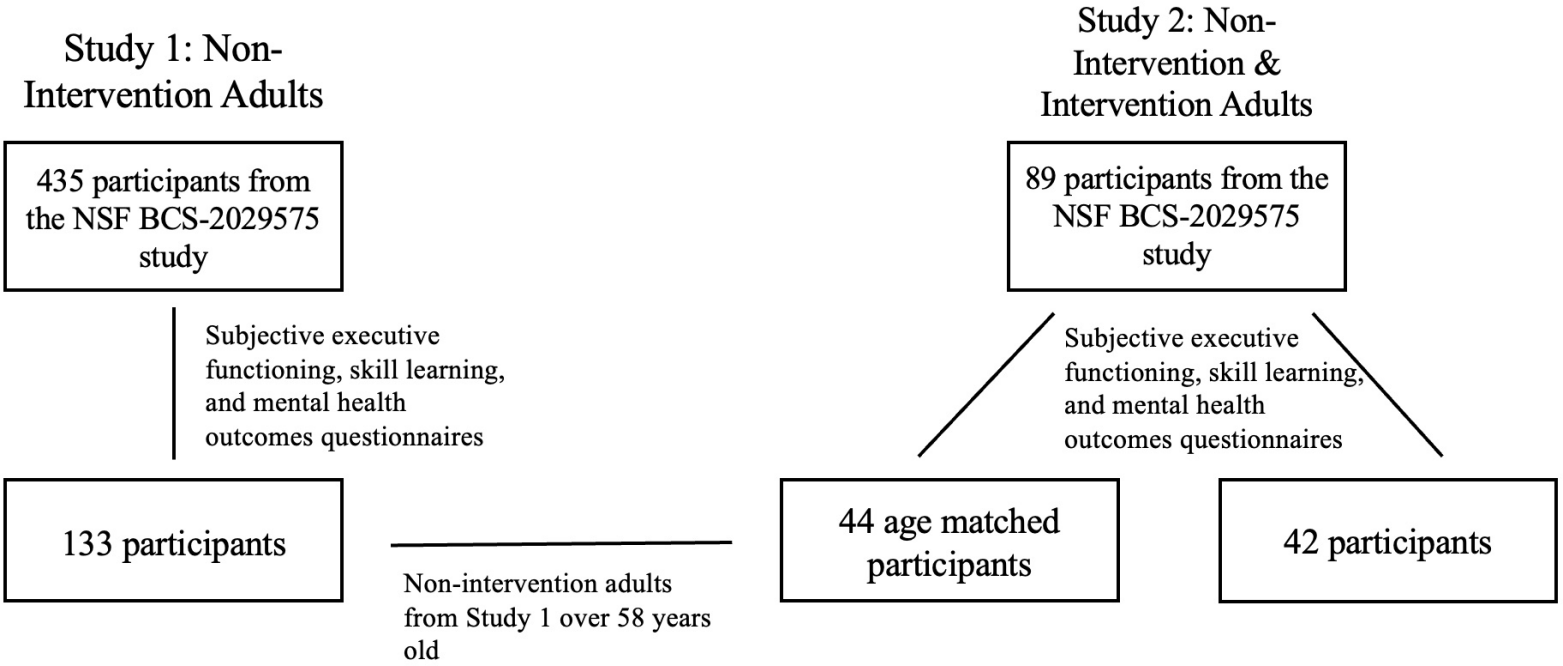
Participants included for Study 1 and Study 2.

Data were collected from 435 participants for Study 1. Data from 44 of those participants from Study 1 were used in Study 2, as well as an additional 89 participants for Study 2 who were previously enrolled in the aforementioned intervention (Participants included for Study 1 and Study 2; Fig 1). The present studies investigated whether subjective executive functioning and novel skill learning in adults predicted mental health outcomes across two time points during the COVID-19 pandemic. Therefore, participants who did not complete the measures assessing subjective executive functioning, skill learning, or mental health outcomes (perceived loneliness, depressive symptoms, and overall well-being) were excluded from the present studies. In addition, participants who failed the questionnaire manipulation checks were also excluded from the present studies. Lastly, participants with missing data on the mental health outcomes scales or more than 3 missing responses on the subjective executive functioning scale were excluded from the present studies. Therefore, Study 1 included data collected from 133 non-intervention adults (73% female; *M*_age_ = 46.17, *SD*_age_ = 17.37, range = 19–89 years).

Study 2 participants included non-intervention adults who also participated in Study 1 and were over 58 years of age to match the subset of adults recruited in the prior cognitive learning intervention [36]. The group that previously participated in the intervention (intervention group) included 42 participants who were not included in Study 1 (91% female; *M*_age_ = 71.00, *SD*_age_ = 5.04, range = 60–86 years), and the non-intervention group in Study 2 (all adults from Study 1 over 58 years of age) included 44 participants (78% female; *M*_age_ = 68.48, *SD*_age_ = 7.57, range = 58–89 years). All participants in the intervention group were enrolled in a cognitive learning intervention prior to the COVID-19 pandemic. Sociodemographic characteristics of the participants are included in Table 1.

**Table 1:** Sociodemographic characteristics from all participants included during Time Point 1.

	Study 1	Study 2	
		Non-Intervention	Intervention
	*n* = 133 (19–89)	*n* = 44 (58–89)	*n* = 42 (58–86)
Characteristics	Mean (*SD*)/%	Mean (*SD*)/%	Mean (*SD*)/%
Age	46.17 (17.37)	68.48 (7.57)	71.00 (5.04)
Gender			
Female	73%	78%	91%
Ethnicity			
Hispanic or Latino	31%	18%	5%
Non-Hispanic or Latino	67%	79%	95%
Prefer not to respond	2%	3%	0%
Race			
White	60%	75%	74%
Black/ African American	7%	8%	10%
Native Hawaiian/ Other Pacific Islander	1%	0%	0%
Asian	10%	8%	2%
American Indian/ Alaskan Native	2%	1%	0%
More than one race	7%	7%	7%
Prefer not to respond	4%	0%	2%
Education			
Professional degree	8%	8%	7%
Master’s degree	31%	22%	4%
Bachelor’s degree	33%	29%	21%
Associate degree	13%	27%	5%
High school diploma	15%	13%	19%

Participants for the present studies were recruited through a combination of social media, phone calls, flyers, and email outreach. Additionally, adults in a database who had previously agreed to be contacted for future studies were contacted via email or phone to participate in this study. Individuals who had participated in the aforementioned intervention and consented to future contact also were invited to complete the survey. All participants provided informed consent, and all data were collected using Qualtrics. Inclusion criteria included: 1) currently reside in Riverside County and San Bernardino County, 2) be fluent in English, and 3) have normal or corrected-to-normal vision. All participants denied having a prior history of Alzheimer’s Disease, dementia, or diagnosed mild cognitive impairment (MCI). During data collection, the first 100 participants in each age group (younger adults 18 – 35, middle aged adults 36 – 64, and older adults 65+) were compensated $20 for completing the study. Subsequent participants (66 adults between the ages of 18–39 years old) were encouraged to complete the survey without immediate compensation and were informed they would be included in follow-up waves of data collection with compensation.

We restricted our data collection to participants in this region for three main reasons: 1) diversity of participants, 2) conservative physical distancing restrictions compared with other Southern California counties, and 3) extreme shortage of healthcare professionals. Approximately 2.41 million individuals reside in Riverside County where 50% of those residents identify as Hispanic, 10% do not have a computer in their home, and 15% do not have internet in their home. Riverside County also was considered one of the most conservative counties in California with respect to the COVID-19 physical distancing restrictions during data collection (Johnson, 2020), and California was one of the first states to impose physical distancing restrictions in the US. Finally, healthcare in Riverside County is currently considered one of the worst in the state of California where the physician to patient ratio is almost three times less than what is recommended by the World Health Organization [WHO.

Data collection for the first timepoint spanned between June 12^th^, 2020, to July 17^th^, 2020. At the start of data collection for the Timepoint 1, the physical distancing restrictions in the State of California were less restrictive than at the initial stage of the pandemic. Businesses within the beauty industry and places of worship were allowed to resume operation at a limited capacity. In addition, celebratory gatherings, such as weddings, were allowed to resume as long as COVID-19 safety regulations were followed (e.g., social distancing, mask requirements, 25% limited capacity). At the start of data collection, Riverside County, which represents over half the population of individuals living in the Inland Empire (2.41 million residents), which consists of Riverside County and San Bernardino County, had approximately 10,000 confirmed COVID-19 cases. However, at the end of data collection (July 13^th^, 2020), Riverside County had approximately 29,000 confirmed COVID-19 cases. Therefore, businesses and places of worship were required to once again follow strict regulations. These regulations included closures of fitness centers, beauty salons, and places of worship if they were unable to provide their services in outdoor spaces.

To minimize the risk of bot responses, attention check questions (e.g., “To confirm you are engaged, please select 7.”) were randomly embedded throughout the questionnaire. The required number varied across different instances of the attention check to ensure attentiveness and reduce the likelihood of automated or inattentive responses. In addition, participants who completed wave 1 and consented for participating in future waves were personally emailed with a questionnaire link as an attempt to limit bot responders. Data collection for wave 2 took place between March 1, 2021, and April 20, 2021. The same set of questionnaires was administered at both timepoints (see Measures). Only participants who completed all measures relevant to the current study at both timepoints, as part of a larger longitudinal project, were included in the subsequent analyses.

### Procedures

Intervention and non-intervention participants (both Studies 1 and 2) were recruited via social media, phone, fliers, or email messages. All participants were provided a brief description of the study and told it would take approximately 1.5 hours to complete. Following informed consent, participants were asked to complete a series of questionnaires. Participants were given the option to complete the survey independently via a computer or mobile device on Qualtrics or have a researcher assist them with completing the survey over the phone by verbally stating their answers. One participant requested to complete the survey over the phone with a research assistant.

### Measures (studies 1 and 2)

#### Perceived loneliness.

The revised UCLA Loneliness Scale [37] assessed individuals’ feelings of perceived loneliness (α = .95). Participants were asked to rate 20 items on a scale from 1 (*strongly disagree*) to 7 (*strongly agree*). An example item is “I lack companionship.” The scores of each item were summed to create a loneliness scale ranging from 20 – 140, where higher scores indicated increased feelings of perceived loneliness (Table 2). Nine items on the scale were required to be reverse scored.

**Table 2:** Predictor and outcome variables descriptive statistics from time point 1.

	Study 1	Study 2	
		Non-Intervention	Intervention
Characteristics	Mean (*SD*)	Mean (*SD*)	Mean (*SD*)
Perceived Loneliness	51.37 (23.94)	43.52 (21.47)	42.35 (23.36)
Depressive Symptoms	31.14 (17.24)	24.85 (13.92)	21.60 (1.14)
Overall Well-being	5.26 (1.06)	5.54 (0.85)	5.40 (1.14)
Subjective executive functioning	129.43 (58.89)	110.84 (49.60)	102.65 (52.39)
Learning new behaviors	7.31 (9.23)	5.37 (5.57)	9.39 (11.79)
Tolerable score	6.28 (2.44)	6.34 (2.55)	7.14 (2.26)
Emotional closeness	8.17 (2.89)	8.43 (3.01)	7.67 (3.17)

#### Depressive symptoms.

We assessed self-reported depressive symptoms with the Patient Health Questionnaire (PHQ-9; [38]). Participants were asked to rate how bothered they were by nine problems (e.g., “feeling down, depressed, or hopeless”) over the last two weeks on a scale from 1 (*not at all*) to 10 (*nearly every day*). The scores of each item were summed to create a score ranging from 9 – 90, where higher scores indicated increased feelings of depressive symptoms (α = .89; Table 2).

#### Overall well-being.

General well-being was assessed with the self-report PERMA-Profiler questionnaire [39]. The questionnaire included 23 items and measured positive emotions, negative emotions, engagement, relationship, meaning, accomplishment, and health. Participants are asked to rate each statement a scale from 1 (*very low*) to 7 (*very high*). We averaged responses to the positive emotions, engagement, relationships, meaning, and accomplishment subscales, then summed the subscale scores to compute an overall well-being score ranging from 12 – 84 (α = .91; Table 2). Higher scores indicated better overall well-being.

#### Subjective executive functioning.

Participants were asked to complete the Behavior Rating Inventory of Executive Function – Adult (BRIEF-A; [40]), a self-report scale that assessed multiple domains under the executive functioning umbrella for adults between the ages of 18 and 90-years-old. The subscales of the BRIEF-A scale used in the present studies included the inhibit, self-monitor, shift, initiate, working memory, plan/organize, organization of materials, and task monitor subscales. Therefore, the BRIEF-A in the present studies consisted of 66 items where participants were asked to rate each item on the scale from 1 (*never*) to 7 (*very often*). An example item is “I forget instructions easily.” The scores of the eight non-overlapping subscales were added to reflect an overall global executive composite score (α = .96; Table 2).

#### New skills and behaviors.

Participants were asked to report the number of hours they spent learning a new skill or behavior during the pandemic from a scale of 0 hours to 50 hours (Table 2). Then, participants were asked to report how tolerable learning a new skill or behavior made physical distancing restrictions (tolerable score) from a scale of 1 (*not tolerable at all*) to 10 (*extremely tolerable*). The hours spent learning a new skill or behavior and the tolerable score were used as independent predictors of outcome variables in data analysis.

#### Emotional closeness.

Emotional closeness was assessed with questions specific to individuals’ housemates. First, participants were asked to report the number of individuals with whom they currently reside. If participants reported living in the same household as others, then they were asked to rate how emotionally close they felt to those individuals they lived with on a scale from 0 (*not close at all*) to 10 (*extremely close*) (Table 2). The emotional closeness score was considered only for individuals who indicated that they were living with others in the same household. Participants (*N* = 23) who indicated that they were living alone but reported an emotional closeness score greater than zero were excluded from all analyses.

#### Socio-demographic information.

Participants answered additional questions assessing socio-demographic factors such as race/ethnicity, sex, total family income, highest level of education, retirement status, and marital status.

### Data analysis (studies 1 and 2)

A linear mixed-effects regression model was conducted for each outcome variable (perceived loneliness, depression, and overall well-being). We included the following predictors: subjective executive functioning, hours spent learning a new skill or behavior during the pandemic, how tolerable learning a new skill or behavior made physical distancing restrictions (i.e., tolerable score), age, and the time point during the COVID-19 pandemic in which the data was collected. As control variables, we included emotional closeness score, and socio-demographic variables, such as age and sex.

Fixed effects represented the “average participant,” and the random effects represented the difference between a particular participant from the average participant. The normality assumption was checked. Wherever it was not satisfied, we performed transformations to the outcome variable (details below). During our analyses, we started with the model that included all predictors/control variables along with all possible two- and three-way interactions among all predictors/control variables. These variables and their interactions were systematically removed to find the optimal model, that is, the model with the smallest Akaike Information Criterion (AIC). To reduce the risk of overfitting, this model selection process was guided both by statistical fit (AIC) and theoretical justification from prior research. Only interaction terms that were conceptually meaningful and improved model performance were retained in the final model. In the model, sex (male and female, with four “no answer” responses that were excluded from all analyses), and time point (first wave of data collection that took place between June 12^th^, 2020, to July 17^th^, 2020 or second wave of data collection that took place between March 1st 2021 to May 24th 2021) were categorical variables. The continuous variables were age, subjective executive functioning, hours spent learning a new skill or behavior during the pandemic, how tolerable learning a new skill or behavior made physical distancing restrictions, and emotional closeness score. For simplicity and clarity, only significant and marginally significant results are reported, and additional details are available upon request.

## Results

### Study 1

Study 1 investigated whether 1) better subjective executive functioning predicted better mental health (less perceived loneliness, less depression, and overall better well-being) during the pandemic across all non-intervention adults, 2) age would predict worse mental health (increased feelings of perceived loneliness and depression, and worse overall well-being), 3) learning a new skill during the pandemic predicted better mental health (less perceived loneliness, less depression, and overall better well-being), and 4) time point during the COVID-19 pandemic predicted mental health outcomes. A post hoc power analysis using G*Power indicated that with our sample size for Study 1 (N = 133) and α = 0.05, the study had 80% power to detect a small-to-medium effect size (f^2^ = 0.08) for a model with 7 predictors, suggesting that the model was powdered to detect small-to-medium associations between the predictor variables and mental health outcomes during the pandemic.

#### Model 1: predicting perceived loneliness in all non-intervention adults.

The normality assumption was met for the first linear mixed-effects regression model with perceived loneliness as the outcome measure (Table 3). Results indicated there was a significant main effect of time point on perceived loneliness (*p* < .001) where wave 1 was the reference group. From the first to the second time point, perceived loneliness increased by 7.12 units. We also found a significant main effect of subjective executive functioning (*p* < .001) and emotional closeness scores (*p* = .007) on perceived loneliness. Keeping all the other predictors constant, as subjective executive functioning increased by one (i.e., became worse), perceived loneliness was estimated to increase by a unit of 0.09. Similarly, all the other predictors constant, as emotional closeness scores increased by one, perceived loneliness was estimated to decrease by 1.35 units.

**Table 3:** Study 1: results from the linear mixed-effects model predicting perceived loneliness.

Predictor	*β* Estimate	*SE*	Cohen’s *f*^*2*^	df	Unadjusted *p*-value
Age	-0.17	0.11	0.04	61	0.135
Sex - Female^1^	-0.07	0.05	0.02	132	0.181
Time Point^2^	7.12	1.70	17.55	1	< 0.001^**^
Subjective Executive Functioning (EF)	0.09	0.03	0.15	61	< 0.001^**^
Tolerable Score	-0.62	0.48	0.03	61	0.206
Emotional Closeness Score	-1.35	0.48	0.13	61	0.007^*^
Learning new behavior	0.09	0.13	0.01	61	0.487

^1^Reference Group: Male.

^2^Reference Group: First time point (June 12^th^, 2020 to July 17^th^, 2020).

Note:

**p < .001,

*p < .05.

#### Model 2: predicting depressive symptoms in all non-intervention adults.

The normality assumption was met for the second linear mixed-effects regression model predicting depressive symptoms (Table 4). Results indicated that there was a significant main effect of age on predicting depressive symptoms (*p* = .026). Keeping all the other predictors constant, as age increased by one unit, depressive symptoms were estimated to decrease by 0.21 units. In addition, there was a significant main effect of subjective executive functioning (*p* < .001) where subjective executive functioning increased by one (i.e., became worse), depressive symptoms were estimated to increase by a unit of 0.14 when all other predictors were kept constant. Lastly, the results indicated there was a significant main effect of hours spent learning a new skill or behavior on predicting depressive symptoms (*p* = .048). Keeping all predictors constant, as the time spent learning a new skill or behavior increased by one unit, depressive symptoms also increased by a unit of 0.51.

**Table 4:** Study 1: results from the linear mixed-effects model predicting depressive symptoms.

Predictors	*β* Estimate	*SE*	Cohen’s *f*^*2*^	df	Unadjusted *p*-value
Age	-0.21	0.07	0.15	61	0.026^*^
Sex - Female^1^	1.51	2.32	0.01	132	0.518
Time Point^2^	2.76	2.00	1.91	1	0.172
Subjective Executive Functioning (EF)	0.14	0.02	0.80	61	< 0.001^**^
Tolerable Score	0.05	0.38	0.01	61	0.898
Emotional closeness score	-0.41	0.34	0.02	61	0.220
Learning new behavior	0.51	0.25	0.07	61	0.048^*^
Interactions					
Time Point X Learning new behavior	-0.37	0.19	0.06	61	0.064

^1^Reference Group: Male.

^2^Reference Group: First time point (June 12^th^, 2020 to July 17^th^, 2020).

Note:

**p < .001,

*p < .05.

The results also indicated a marginally significant interaction between time point and time spent learning a new skill or behavior (*p* = .064). Keeping all the other predictors constant including the number of hours spent learning a new skill or behavior, it was predicted that depressive symptoms during wave 2 decreased by 3.13 units. Similarly, keeping all the other predictors constant including time point, as the time spent learning a new skill or behavior increased by one unit it was predicted that depressive symptoms were estimated to decrease by 0.88 units.

#### Model 3: predicting overall well-being in all non-intervention adults.

The normality assumption was met for the third linear mixed-effects regression model predicting overall well-being (Table 5). Results indicated there was a significant main effect of time point on overall well-being (*p* < .001) where wave 1 was the reference group. From the first to the second time point, overall well-being decreased by 4.89 units. In addition, we found a significant main effect of subjective executive functioning (*p* < .001). Keeping all the other predictors constant, as subjective executive functioning increased by one (i.e., became worse), overall well-being was estimated to decrease by a unit of 0.07. Lastly, we found a significant main effect of emotional closeness scores (*p* < .001) on predicting overall well-being. When all the other predictors were kept constant, as emotional closeness scores increased by one, overall well-being was estimated to increase by 1.23 units.

**Table 5:** Study 1: results from the linear mixed-effects model predicting overall well-being.

Predictors	*β* Estimate	*SE*	Cohen’s *f*^*2*^	df	Unadjusted *p*-value
Age	0.08	0.07	0.02	61	0.272
Sex - Female^1^	3.44	2.65	0.01	129	0.197
Time Point^2^	-4.89	1.32	13.69	1	< 0.001^**^
Subjective Executive Functioning (EF)	-0.07	0.02	0.20	61	< 0.001^**^
Tolerable Score	0.51	0.36	0.03	61	0.269
Emotionally closeness score	1.23	0.34	0.22	61	0.001^*^
Learning new behavior	0.13	0.10	0.03	61	0.195

^1^Reference Group: Male.

^2^Reference Group: First time point (June 12^th^, 2020 to July 17^th^, 2020).

Note:

**p < .001,

*p < .05.

### Study 2

Study 2 investigated whether better subjective executive functioning predicted less perceived loneliness, less depression, and overall better well-being during the pandemic for adults over the age of 58 who were enrolled in a cognitive learning intervention prior to the pandemic, compared with 58 + year old adults from Study 1 who did not participate in the intervention. In addition, Study 2 investigated whether learning new skills during the pandemic predicted less perceived loneliness, less depression, and overall better well-being during the pandemic and if time-point during the COVID-19 pandemic predicted mental health outcomes. Following Study 1, three linear mixed-effects regression models were conducted. A post hoc power analysis using G*Power indicated that with our sample size for Study 2 (N = 86), α = 0.05, and 8 predictors in the model, the study had 80% power to detect an effect size of *f*2 = 0.124. This suggests that Study 2 was powered to detect medium-sized effects of the predictors on the outcome variables.

#### Model 1: predicting perceived loneliness in intervention vs. non-intervention adults (58+).

The first linear mixed-effects regression model predicting perceived loneliness in the intervention vs. non-intervention groups (Table 6) included a log-transformation of the outcome variable to satisfy the normality assumption. Results indicated there was a significant main effect of age (*p* = .014) on predicting perceived loneliness. Keeping all the other predictors constant, as age increased by one, perceived loneliness was estimated to decrease by 2.30%. In addition, similar to study 1, we found a significant main effect of subjective executive functioning (*p* = .008) on perceived loneliness. Keeping all the other predictors constant, as subjective executive functioning increased by one (i.e., became worse), perceived loneliness was estimated to increase by 0.30%. There was also a marginally significant main effect of emotional closeness score on predicting perceived loneliness (p = 0.076). Keeping all the other predictors constant, as emotional closeness scores increased by one, perceived loneliness was estimated to decrease by 2.70%.

**Table 6:** Study 2: results from the linear mixed-effects model predicting perceived loneliness.

Predictors	*β* Estimate	*SE*	Cohen’s *f*^*2*^	df	Unadjusted *p*-value
Age	-0.02	0.01	0.36	11	0.014^*^
Sex - Female^1^	-0.17	0.15	0.03	51	0.239
Time Point^2^	0.26	0.10	6.76	1	0.022^*^
Intervention	-0.06	0.10	0.03	11	0.552
Subjective Executive Functioning (EF)	0.01	0.00	0.57	11	0.008^*^
Tolerable Score	-0.02	0.02	0.09	11	0.322
Emotionally closeness score	-0.03	0.01	0.82	11	0.076
Learning new behavior	0.04	0.01	1.46	11	0.008^*^
Interactions					
Time Point X Learning new behavior	-0.02	0.01	0.36	11	0.042^*^

^1^Reference Group: Male.

^2^Reference Group: First time point (June 12^th^, 2020 to July 17^th^, 2020).

Note:

**p < .001,

*p < .05.

There was significant interaction between time point and time spent learning a new skill or behavior (*p* = .042) on predicting perceived loneliness. Keeping all the other predictors constant including the number of hours spent learning a new skill or behavior, it was estimated that perceived loneliness during wave 2 decreased by 29.00%. Similarly, keeping all the other predictors constant including time point, as the time spent learning a new skill or behavior increased by one unit it was predicted that perceived loneliness decreased by 5.40%

#### Model 2: predicting depressive symptoms in intervention vs. non-intervention adults (58+).

The normality assumption was met for the second regression model predicting depressive symptoms in the intervention vs. non-intervention groups (Table 7). Results indicated that there was a significant main effect of age (*p* = .030) on predicting depressive symptoms. Keeping all the other predictors constant, as age increased by one, depressive symptoms was estimated to decrease by a unit of 0.47. In addition, there was a significant main effect of subjective executive functioning (*p* < .001). Keeping all the other predictors constant, as subjective executive functioning increased by one (i.e., became worse), depressive symptoms were estimated to increase by a unit of 0.12. Lastly, there was a significant main effect of emotional closeness score on predicting depressive symptoms (p = 0.005). Keeping all the other predictors constant, as emotional closeness scores increased by one, depressive symptoms was estimated to decrease by 1.17 units.

**Table 7:** Study 2: results from the linear mixed-effects model predicting depressive symptoms.

Predictors	*β* Estimate	*SE*	Cohen’s *f*^*2*^	df	Unadjusted *p*-value
Age	-0.47	0.19	0.51	12	0.030^*^
Sex - Female^1^	-5.21	3.51	0.04	53	0.143
Time Point^2^	-12.56	5.97	4.41	1	0.057
Intervention	-3.17	2.28	0.16	12	0.207
Subjective Executive Functioning (EF)	0.12	0.02	3.00	12	<0.001^**^
Tolerable Score	-3.55	1.31	0.61	12	0.019^*^
Emotionally closeness score	-1.17	0.34	0.99	12	0.005^*^
Learning new behavior	1.15	0.31	1.15	12	0.003^*^
Interactions					
Time Point X Learning new behavior	-0.71	0.23	0.00	10112	0.042^*^
Subjective EF Time Point X Tolerable Score	0.003	0.89	0.00	10112	0.080

^1^Reference Group: Male.

^2^Reference Group: First time point (June 12^th^, 2020 to July 17^th^, 2020).

Note:

**p < .001,

*p < .05

There was significant interaction between time point and time spent learning a new skill or behavior (*p* = .009) on predicting depressive symptoms. Keeping all the other predictors constant including the number of hours spent learning a new skill or behavior, it was predicted that depressive symptoms during wave 2 increased by 11.85 units. Similarly, keeping all the other predictors constant including time point, as the time spent learning a new skill or behavior increased by one unit it was predicted that depressive symptoms decreased by 1.86 units.

There was also a significant interaction between time point and the tolerable score (*p* = .005). Keeping all the other predictors constant including the tolerable score, it was predicted that depressive symptoms increased by 15.58 units between the two time points. Similarly, keeping all the other predictors constant including time point, as the tolerable score increased by one unit it was predicted that depressive symptoms increased by 6.57 units.

#### Model 3: predicting overall well-being in intervention vs. non-intervention adults (58+).

The normality assumption was met for the third linear mixed-effects regression model predicting overall well-being in the intervention vs. non-intervention groups (Table 8). Results indicated that there was a significant main effect of age (*p* = .025) on predicting overall well-being. Keeping all the other predictors constant, as age increased by one year, overall well-being was estimated to increase by a unit of 0.74. In addition, there was a significant main effect of subjective executive functioning (*p* = .013). Keeping all the other predictors constant, as subjective executive functioning increased by one (i.e., became worse), overall well-being was estimated to decrease by unit of 0.10. There was also a marginally significant main effect of intervention status (*p* = 0.063) and emotional closeness score (*p* = 0.062) on predicting overall well-being. Keeping all the other predictors constant, those who participated in the intervention were estimated to have overall well-being decreased by 18.74 units. In addition, when all other predictors were kept constant, as emotional closeness scores increased by one, overall well-being was estimated to increase by 1.08 units.

**Table 8:** Study 2: results from the linear mixed-effects model predicting overall well-being.

Predictors	*β* Estimate	*SE*	Cohen’s *f*^*2*^	df	Unadjusted p-value
Age	0.74	0.28	0.69	10	0.025^*^
Sex - Female^1^	5.86	5.03	0.03	51	0.249
Time Point^2^	-6.49	3.69	3.10	1	0.109
Intervention	-18.74	8.98	0.43	10	0.063
Subjective Executive Functioning (EF)	-0.10	0.03	1.11	10	0.013^*^
Tolerable Score	2.78	0.94	0.87	10	0.015^*^
Emotionally closeness score	1.08	0.51	0.44	10	0.062
Learning new behavior	1.80	0.93	0.37	10	0.083
Interactions					
Time Point X Intervention	13.21	6.00	0.48	10	0.052
Tolerable Score X Learning new behavior	-0.28	0.12	0.54	10	0.042^*^

^1^Reference Group: Male.

^2^Reference Group: First time point (June 12^th^, 2020 to July 17^th^, 2020).

Note:

**p < .001,

*p < .05.

There was a significant interaction between the tolerable score and the number of hours spent learning a new skill or behavior (*p* = .042). Keeping all the other predictors fixed, including the number of hours spent learning a new skill or behavior, as the tolerable score increased by one unit, the overall well-being score was estimated to decrease by 3.06 units. Similarly, keeping all the other predictors fixed, including the tolerable score, for every hour spent learning a new skill or behavior, overall well-being scores were estimated to decrease by 2.08 units.

There was also a marginally significant interaction between time point and intervention status (*p* = .052). Keeping all the other predictors constant including the intervention status, it was predicted that overall well-being during wave 1 increased by 19.70 units. Similarly, keeping all the other predictors constant including time point, it was predicted that overall well-being for the non-intervention group increased by 31.96.

## Discussion

Study 1 investigated whether better subjective cognitive abilities (i.e., executive functioning) and increased novel skill learning predicted less perceived loneliness, less depressive symptoms, and better overall well-being during the COVID-19 pandemic. Study 2 investigated these relationships in a sample of adults who participated in a cognitive learning intervention prior to the pandemic that increased objective executive functioning [21], compared with adults who did not participate in the intervention (i.e., middle-aged and older adults from Study 1).

In general, there were many similarities between our findings from Studies 1 and 2. In both studies, we found that worse subjective executive functioning predicted worse overall mental health, which is consistent with prior studies both before and during the pandemic [11–13]. In addition, across both studies, we found that experiencing a sense of emotional closeness, or interpersonal ties, during the COVID-19 pandemic positively impacted overall well-being. This result is in line with prior studies that suggest that close interpersonal ties or relationships can positively impact mental health [41,42]. Furthermore, in Study 2 we found a significant interaction between Timepoint and time spent learning a new skill or behavior on mental health outcomes across adults over the age 58. Specifically, perceived loneliness and depressive symptoms decreased during the second timepoint if participants spent more time learning a new skill or behavior. These results are in line with prior studies suggesting that engaging in leisure activities, which can include learning new skills, may be beneficial for mental health outcomes (26) and could improve overall mental health [22,24,25]. Lastly, across both Studies 1 and 2, we found that age may have served as a protective factor: as age increased, participants reported fewer depressive symptoms and lower levels of perceived loneliness. Additionally, in Study 2, older participants reported higher overall well-being. These results were inconsistent with our predictions and some pre-pandemic literature on mental health outcomes across age [[43–45], although see Carstensen et al., 2020]. However, our findings align with studies conducted during the pandemic showing older adults’ maintained well-being.

Prior to the pandemic, learning new skills has been shown to benefit both cognitive and mental health outcomes [21,22,24,25]. Interestingly, adults in the present studies who spent more time learning a new skill or behavior during the second timepoint of data collection in the pandemic seemed to experience overall better mental health outcomes. These results provide an approach to understanding the role of novel skill learning on mental health, especially during different timepoints of the COVID-19 pandemic. These results and results observed in prior studies during the COVID-19 pandemic [9,10,27,28] and longitudinal studies examining changes in mental health outcomes from pre-COVID-19 pandemic to the first time point of data collection during the pandemic [29–32] contribute to the mixed findings in the literature to date regarding the impact of the COVID-19 pandemic on mental health. While the results in the present studies suggest that spending more time learning a new skill or behavior may have acted as a protective factor for mental health during the second timepoint of data collection during the COVID-19 pandemic, prior studies have presented mixed findings regarding mental health outcomes during the pandemic [9,10,27,29–32]. For instance, studies assessing mental health during a single timepoint in the first few months of the COVID-19 pandemic have reported worse mental health outcomes for younger adults than older adults [9,10]. However, one longitudinal study during the COVID-19 pandemic suggested that younger adults’ may experience declines in their mental health during the early stages of the pandemic when compared to older adults, but during later stages of the pandemic older adults’ mental health may become worse than that of younger adults [27].

While the results of the present studies did not find an interaction between timepoint and age, in Study 1 we found that as age increased, depressive symptoms decreased during the COVID-19 pandemic. Similarly, in Study 2 we found that as age increased, perceived loneliness and depressive symptoms decreased, and overall well-being increased. While these results are inconsistent with the well-documented pre-pandemic studies investigating the association between mental health and age [11–13], they are in line with studies investing mental health outcome during the pandemic suggesting that younger adults may have experienced worse mental health outcomes when compared to older adults during the COVID-19 pandemic [9,10]. One possible explanation is that older adults may be engaging more in the age-related positivity effect, which refers to the tendency for older adults, compared to younger adults, to preferentially attend to and remember positive over negative information [see 45]. The positivity effect may serve as a psychological buffer, helping older adults maintain better mental health outcomes by dampening the impact of negative information, such as COVID-19-related news, during a time of crisis [46]. In addition, younger adults may have lost more opportunities for mental growth, such as reduced or cancelled classes for undergraduates, leading to, for example, anxiety about future career prospects (see [47]). This loss of learning opportunities may have negatively impacted the older adults in our intervention group as well.

While marginally significant, we also found that in Study 2, participants from the cognitive learning intervention were more likely to report worse overall well-being during the COVID-19 pandemic than those who did not participant in the cognitive learning intervention. In addition, we found a marginally significant interaction between timepoint and intervention status where those who participated in the cognitive learning intervention reported worse overall well-being during the first timepoint of data collection during the pandemic. The present studies provides some evidence for a more nuanced approach to understanding cognitive abilities, novel skill learning, and mental health during the pandemic. In particular, adults who participated in the prior cognitive learning intervention may have had increased susceptibility to the negative effects of the physical distancing restrictions on their mental health. The sudden loss of in-person learning opportunities for these participants could have been perceived as substantial (perhaps similar to college students), especially since during the intervention, the intervention participants engaged in 15 hours a week of learning activities on average. The intervention participants also had a loss of social opportunities associated with participating in the in-person intervention. For adults who did not previously participate in this cognitive learning intervention, the relationship between worse overall well-being was attenuated.

### Limitations and future directions

There are some limitations to note in the present studies. First, the use of self-report measures, such as self-reported EF, perceived loneliness, depressive symptoms, well-being, and time spent learning a new skill or behavior could lead to a the social desirability bias (Paulhus, 1984), where individuals typically want to appear more favorable when responding to self-report survives. However, we can rule this bias out in terms of subjective EF given that subjective and objective measures of cognitive functioning are associated [17,18], our measure of subjective EF may provide an accurate representation of participants’ actual EF. Despite this, future studies should consider including objective, in addition to subjective, measures of cognitive functioning. Lastly, our findings on learning adaptation may relate to stress adaptation in responding to the effects of the pandemic (e.g., [48], but future studies need to clarify the similarities and differences of learning adaptation and stress adaptation.

## Conclusions

The present studies found that worse subjective executive functioning (EF) during the COVID-19 pandemic predicted worse mental health across adulthood, which is in line with studies prior to the pandemic [11–13]. In addition, the present studies found that participants who spent more time learning a new skill or behavior during the second timepoint of data collection of the pandemic experienced overall better mental health outcomes. This finding is consistent with prior studies showing that participating in leisure activities benefits overall mental health [22,24,25]. Our findings highlight the importance of considering cognitive abilities and novel skill learning (perhaps related to adaptability) on mental health during a highly dynamic period.
